# Effects of Cone Connexin-36 Disruption on Light Adaptation and Circadian Regulation of the Photopic ERG

**DOI:** 10.1167/iovs.61.6.24

**Published:** 2020-06-12

**Authors:** Shuo Zhang, Polina Lyuboslavsky, Jendayi Azeezah Dixon, Micah A. Chrenek, Jana T. Sellers, Jessica M. Hamm, Christophe P. Ribelayga, Zhijing Zhang, Yun Z. Le, P. Michael Iuvone

**Affiliations:** 1 Department of Ophthalmology, Emory University, School of Medicine, Atlanta, Georgia, United States; 2 Beijing Tongren Eye Center, Beijing Tongren Hospital, Beijing Key Laboratory of Ophthalmology and Visual Sciences, Capital Medical University, Beijing, China; 3 Department of Pharmacology, Emory University, School of Medicine, Atlanta, Georgia, United States; 4 Ruiz Department of Ophthalmology & Visual Science, McGovern Medical School, The University of Texas Health Science Center at Houston, Houston, Texas, United States; 5 Departments of Medicine, Cell Biology, and Ophthalmology and Harold Hamm Diabetes Center, University of Oklahoma Health Sciences Center, Oklahoma City, Oklahoma, United States

**Keywords:** connexin-36, circadian rhythm, light adaptation, electroretinography, gap junctions, photoreceptors

## Abstract

**Purpose:**

The present study tested the hypothesis that connexin-36 (Cx36) and gap junctions between photoreceptor cells contribute to the circadian rhythm of the photopic electroretinogram (ERG) b-wave amplitude.

**Methods:**

Cone-specific disruption of Cx36 was obtained in mice with a floxed *Gjd2* gene and human red/green pigment promoter (HRGP)-driven Cre recombinase. Standard ERG, spectral-domain optical coherence tomography (SD-OCT) and histochemical methods were used.

**Results:**

*HRGP^cre^Gjd2^fl/fl^* mice had a selective reduction in Cx36 protein in the outer plexiform layer; no reduction in Cx36 was observed in the inner plexiform layer. Cx36 disruption had no effect on the number of cones, the thickness of the photoreceptor layer, or the scotopic ERG responses. However, there was a reduction of the photopic ERG circadian rhythm, with b-wave amplitudes in the day and the night locked in the daytime, light-adapted state. In *HRGP^cre^Gjd2^+/+^*and *Gjd2^fl/fl^* controls, the circadian rhythm of light-adapted ERG persisted, similar to that in wild type mice.

**Conclusions:**

Cx36 regulation contributes to the circadian rhythm of light-adapted ERG; in the absence of photoreceptor gap junctions, mice appear to be in a fully light-adapted state regardless of the time of day. The higher amplitudes and reduced circadian regulation of the b-wave of *HRGP^cre^Gjd2^fl/fl^* mice may be due to increased synaptic strength at the cone to ON bipolar cell synapse due to electrotonic isolation of the terminals lacking gap junctions.

Circadian rhythms are biological rhythms that occur with a period of approximately 24 hours. They are endogenously generated and entrained by environmental stimuli, most notably light.^1^ Circadian rhythms exhibit as cycles of sleep and wakefulness, hormone secretion, blood pressure, body temperature, neuronal activity, and metabolism. Disruption of circadian rhythm genes leads to diseases or disease susceptibility in different tissues, including metabolic (obesity and diabetes), sleep, mood, developmental disorders, and cancer.^2^^,^^3^

Most tissues and cells generate their own intrinsic circadian rhythms, and also receive and respond to the input of circadian signals from the circadian pacemakers located in the brain – the suprachiasmatic nuclei (SCN). However, the retina contains an autonomous circadian clock network that is largely independent of the SCN.^4^ Retinal clocks regulate rhythms of photoreceptor outer segment disc shedding, melatonin synthesis in photoreceptors, dopamine release from amacrine cells, contrast sensitivity mediated by retinal ganglion cells, photopic electroretinogram (ERG) responses, post-translational modifications of proteins, and transcription of circadian clock genes and clock-controlled genes all exhibit rhythmicity. Of particular interest is the light-adapted ERG, which is regulated in a circadian fashion with higher b-wave amplitudes during the subjective day than the subjective night.^5^^,^^6^ Although the mechanism of this rhythm is known to involve the neurotransmitter dopamine^7^ and retinal circadian clock genes,^5^^,^^6^ including *Bmal1* and cryptochromes, the cellular/molecular basis of the rhythm is unknown.

There are five main types of cell junctions. One of them is the gap junction, which forms channels between cells that can conduct ion currents and pass other small molecules. Gap junctions are essential for intercellular communication in most tissues. Gap junction channels consist of two apposed hemi-channels from contiguous cells. In vertebrates, each hemi-channel is formed by six protein subunits of connexin proteins.^8^^,^^9^

There are approximately 20 different connexin isoforms in mammals.^10^^,^^11^ Among the connexin family, connexin36 (Cx36) is mainly expressed in the adult mammalian central nervous system, including the retina, where it forms electrical synapses among cells. The Cx36 protein is encoded by the *Gjd2* gene. In the retina, Cx36 proteins form gap junctions between rod photoreceptors, rod and cone photoreceptors, and many inner retinal neurons forming electrical synapse networks that regulate various types of neuronal plasticity and network adaptation.^9^^,^^12^

Cx36 expression and phosphorylation in the retina has been shown to be regulated by light, dopamine, and circadian clocks.^12^ Cx36 gap junctions between rods and cones are open in darkness and dim light, and closed in bright light. This pathway is thought to provide a secondary pathway for rod signals through cones and cone bipolar cells to retinal ganglion cells.^13^ Given the prominent role of gap junctions in photoreceptor signaling, we explored their potential role in the circadian regulation of light-adapted ERG responses. In the project reported here, we characterized the ERG light responses and retinal morphological changes in mice with cone-specific disruption of *Gjd2*, compared to mice with disruption of the circadian clock gene encoding Bmal1 (*Arntl*) and to wild type mice.

## Methods

### Experimental Animals

*HRGP^cre^* mice, which express Cre recombinase in cone photoreceptors, were described previously.^14^ Ai6 (RCL-ZsGreen) mice [B6.Cg-Gt(ROSA)26Sor^tm6(CAG-ZsGreen1)/Hze^/J], a Cre reporter line,^15^ were obtained from The Jackson Laboratory (Bar Harbor, ME, USA). *Gjd2^fl/fl^* were generated as described in Yao et al.^16^ and generously provided by David Paul, Harvard University. Mice were genotyped by PCR analysis of genomic DNA. Mice of both genders were used at 3 to 6 months of age. They were housed under a 12-hour light – 12 hours of dark cycle of illumination (luminance of 50-100 lux at cage level) with lights on from zeitgeber time (ZT) 0 to 12, and were given food and water ad libitum. All experimental procedures in “dark” conditions were performed under dim red light. Mice were euthanized by CO_2_ asphyxiation followed by cervical dislocation. All procedures were approved by Emory University's Institutional Animal Care and Use Committee and conformed to the guidelines of the National Institutes of Health Guide for the Care and Use of Laboratory Animals and the ARVO Statement for the Use of Animals in Ophthalmic and Vision Research.

### ERG Recordings

To assess retinal light responses, ERG recordings were collected with an LKC UTAS Visual Diagnostic System with BigShot ganzfeld (LKC Technologies), as described by Jackson et al.^7^ with minor modifications. Mice were dark adapted for a minimum of 15 hours, and for as much as 54 hours, as indicated for each experiment. They were anesthetized with an intraperitoneal (IP) injection of a cocktail of ketamine (100 mg/kg) and xylazine (15 mg/kg). Body temperature was maintained at approximately 37 degrees Celsius (°C) using a heating pad (Model TC-1000 Temperature Controller; CWE, Ardmore, PA, USA) fixed on the ERG stage. Topical 0.5% proparacaine HCL eye drops (Alcon Labs, Inc., Fort Worth, TX, USA) were applied and the pupils were dilated with 1% tropicamide ophthalmic solution (Bausch & Lomb, Rochester, NY, USA). Their eyes were kept moist with Refresh Tears (0.5% carboxymethylcellulose sodium) eye drops. Stainless-steel needle electrodes were gently inserted into each cheek to serve as a reference and another one was placed just under the skin of the tail as the ground. The mouse head was positioned in front of a Ganzfeld stimulus. DTL electrodes were placed on the corneal surface of each eye for recording of dark-adapted and light-adapted ERG responses. Recordings were made in the middle of the subjective day (circadian time [CT] 4–8) or the middle of the subjective night (CT 16–20), as described by Jackson et al.^7^

### Spectral Domain-Optical Coherence Tomography 

Fundus photographs and spectral domain-optical coherence tomography (SD-OCT) images of the retina of anesthetized mice were obtained with the Micron IV imaging system (Phoenix Research Labs, Pleasanton, CA, USA). Lubricant eye gel, GenTeal (Alcon, Fort Worth, TX, USA) was applied to the eyes before imaging to keep cornea moist and mice were kept warm on a heating pad. SD-OCT measurements were made of a linearized circle with a diameter of 0.57 mm centered on the optic nerve. The average thickness of the retina and photoreceptor layer was measured using Adobe Photoshop CS6.

After ERG and OCT recordings, mice were injected Antisedan (0.5 mg/kg, IP) to facilitate recovery of anesthesia.

### Immunofluorescence of Cx36

Following euthanasia, eyes were enucleated and the superior cornea of each eye was marked with a surgical cautery pen (Bovie Medical, Clearwater, FL, USA). Eyes were fixed for 1 hour at room temperature in 4% paraformaldehyde (PFA) in PBS, washed twice in PBS, and cryoprotected overnight in 30% sucrose at 4°C. The eyes were frozen in Optimal Cutting Temperature embedding medium and sectioned in a cryostat at 16 µm thickness.

Antigen retrieval was achieved by boiling for 3 minutes in 10 mM citric acid, pH 6. Slides were washed 3 times with deionized water and blocked for 2 hours with 5% normal donkey serum in Tris buffered saline containing 0.1% Tween-20 (TBS-T). Primary antibodies (mouse anti-connexin 36 [MAB3045]: 1:200; Millipore, Burlington, MA, USA; mouse anti-connexin 36 [37-4600]: 1:200; ThermoFisher Scientific, Waltham, MA, USA; rabbit anti-cone arrestin [AB15282]: 1:1500; Abcam, Cambridge, UK) were applied diluted in blocking buffer and incubated overnight at room temperature. Slides were washed 3 times with TBS-T, 10 minutes each, followed by incubation in secondary antibodies (donkey anti-mouse Alexa Fluor 488: 1:1000; Jackson Immunoresearch Laboratories, West Grove, PA, USA; donkey anti-rabbit Alexa Fluor 568: 1:1000; ThermoFisher Scientific) diluted in TBS buffer for 2 hours at room temperature. Following incubation, slides were washed with TBS-T 3 times, 10 minutes each, dried, and cover slipped using mounting medium with DAPI (Sigma, St. Louis, MO, USA).

Images were captured at 20×, 60×, and 240× with a Nikon Eclipse C1 confocal microscope. The number of plaques in the outer plexiform layer (OPL) and inner plexiform layer (IPL) were quantified using Imaris 9.0.2 (Bitplane USA, Concord, MA, USA).

### Immunofluorescence of Cone Arrestin 

Eyes were marked superior using a cautery tool prior to removal from the mouse. The eyes were oriented using this mark during embedding in paraffin, such that they were sectioned in the sagittal plane allowing identification of the superior and inferior retina. Freeze substitution fixed (97% methanol with 3% acetic acid) paraffin sections were deparaffinized and rehydrated through graded ethanol series. Endogenous peroxidases were inactivated with 3% hydrogen peroxide treatment and then blocked with 2.5% donkey serum. Rabbit anti-cone arrestin (CARR) antibody from Millipore cat # AB15282 was used to stain cones. Slides were stained for 1 hour using a 1:500 dilution of the primary antibody. Donkey anti-rabbit-HRP secondary at 1:500 dilution (Invitrogen A16035) for 1 hour and Tyramide-AlexaFluor 488 Superboost kit (ThermoFisher Life Technologies) were used to stain the location of primary antibody binding. Slides were counterstained with propidium iodide (ThermoFisher Life Technologies) and imaged using Nikon C1 confocal imager. Cones numbers were analyzed in single 5 µm sections of both eyes of each mouse and averaged. CARR positive cells were counted in 500 µm regions at the far periphery of the superior and inferior retina in the sections starting where the retina meets the ciliary body, and 500 µm regions on either side of the optic nerve head starting 100 µm from the edge of the optic nerve.

### Statistics

Data are expressed as mean ± standard error of the mean (SEM). Comparison of the 2 groups was achieved by two-tailed Student's *t*-test. Data from more than two groups was analyzed 2-way ANOVA. Multiple comparisons were made using the Holm-Sidak method.

## Results

### Selective Loss of Cx36 in Cone Photoreceptors of *HRGP^cre^Gjd2^fl/fl^* mice

**Figure 1.**
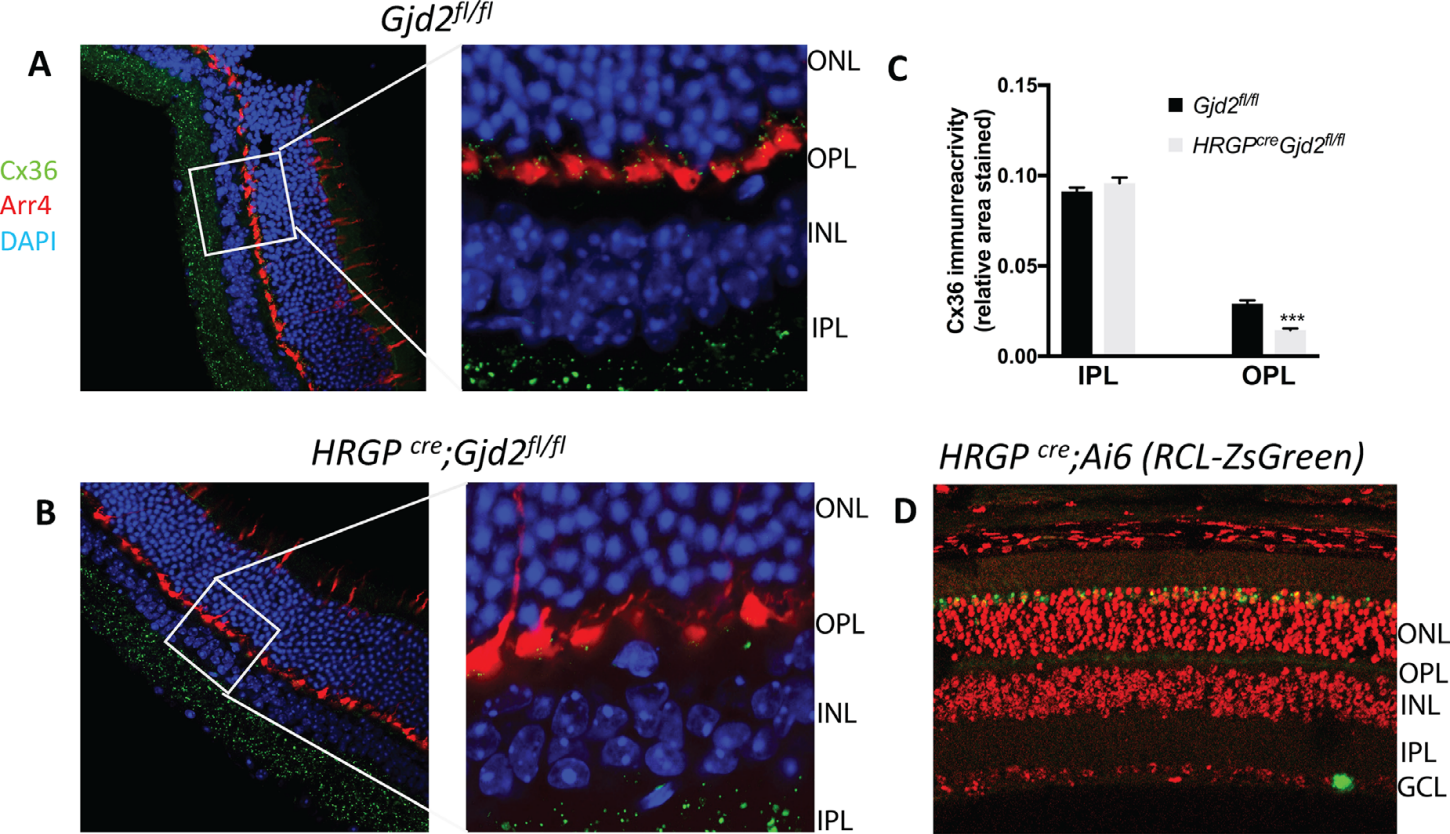
Immunofluorescence staining of retinas showed selective disruption of Cx36 expression in cone photoreceptors in *HRGP^cre^ Gjd2^fl/fl^*. (**A, B**) Cx36 immunoreactive plaques were observed in the IPL and OPL of *Gjd2^fl/fl^* and *HRGP^cre^ Gjd2^fl/fl^* mice (magnification: left 60×, right 240×); see black and white images in Supplementary Figure S1. (**C**) Quantification of Cx36 immunoreactive plaques in retinas of *HRGP^cre^Gjd2^fl/fl^* mice was lower compared to the littermate *Gjd2^fl/fl^* control mice (***P < 0.001, sample size: 6–7 mice / genotype). (**D**) Fluorescence was observed almost exclusively in cone photoreceptors in Ai6 (RCL-ZsGreen) Cre reporter mice. Retinal layers: ONL, outer nuclear layer; OPL, outer plexiform layer; INL, inner nuclear layer; IPL, inner plexiform layer; GCL, ganglion cell layer.

Connexin-36 was localized by immunofluorescence in retinas of *Gjd2^fl/fl^ and HRGP^cre^Gjd2^fl/fl^* mice (Fig. 1 A, B; see Supplementary Fig. S1 for a black and white image). In all the samples, Cx36 immunoreactive plaques were observed in the IPL and OPL. In retinas of *HRGP^Cre^Gjd2^fl/fl^* mice, Cx36 staining was lower compared to the *Gjd2^fl/fl^* littermate control mice in the OPL but not in the IPL (Fig. 1C, Figure S1). Quantification of Cx36 immunoreactive plaques in retinas of *HRGP^cre^Gjd2^fl/fl^* mice and littermate *Gjd2^fl/fl^* control mice confirmed the selective reduction of 49% in the OPL of the cone-specific knockouts, with no difference in the IPL (Fig. 1C).

To assess the expression pattern of *HRGP^cre^* in the retina, mice were bred with Ai6 (RCL-ZsGreen) reporter mice (Fig. 1D). ZsGreen fluorescence was observed almost exclusively in cone photoreceptors. An occasional, unidentified cell was found in the retinal ganglion cell layer, as was observed for the lacZ reporter in the original publication on these mice.^14^ This pattern is consistent with the selective reduction of Cx36 in cones of *HRGP^cre^Gjd2^fl/fl^* mice compared to the floxed control (Fig. 1C).

### ERG Analysis of Cone-Specific Disruption of *Gjd2*

**Figure 2.**
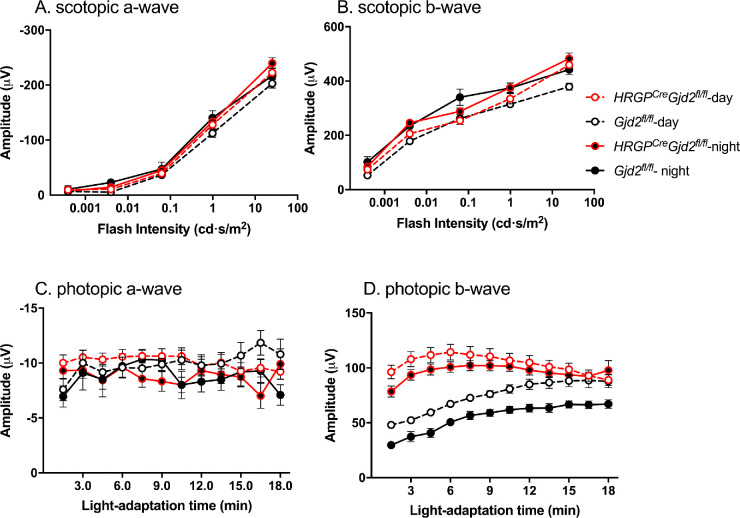
ERG recordings of *HRGP^cre^Gjd2^fl/fl^* mice and littermate *Gjd2^fl/fl^* control mice. (**A, B**) Scotopic ERG responses as a function of flash intensity. No significant differences were observed for time of day or genotype. (**C, D**) Photopic ERG responses as a function of light-adaptation time (protocol 1). **C** Photopic a-wave responses showed no consistent effect of either light-adaptation time or genotype. **D** The amplitude of the circadian rhythm of b-wave amplitude in *HRGP^cre^Gjd2^fl/fl^* mice was reduced compared to that of *Gjd2^fl/fl^* mice, and was significantly higher than controls at light-adaptation times of 1.5 to 12 minutes at both midday and midnight (*P* < 0.01). *Gjd2^fl/fl^* showed a significant effect of light-adaptation time and of time-of-day (*P* < 0.001). Sample sizes: *HRGP^cre^Gjd2^fl/fl^* 18 mice; *Gjd2^fl/fl^* 8 mice. Comparisons of recordings from *HRGP^cre^Gjd2^fl/fl^* mice with wild type and *HRGP^cre^Gjd2^+/+^* control mice are shown in Supplementary Figures S2 and S3.

Dark-adapted and light-adapted ERG responses were analyzed in *HRGP^cre^Gjd2^fl/fl^* mice and all control genotypes (*Gjd2^fl/fl^*, *Gjd2^+/+^*, *HRGP^cre^Gjd2^+/+^*). Mice were dark-adapted beginning at ZT 12, the time of light offset of the light-dark cycle, and analyzed during the middle of the following subsequent subjective day or the subjective night. As shown in Figure 2, there were no circadian differences in a- or b-wave amplitudes of the dark-adapted ERG in either *HRGP^cre^Gjd2^fl/fl^* mice or littermate *Gjd2^fl/fl^* control mice. In addition, there were no differences in dark-adapted a- or b-wave amplitudes between these two genotypes (Fig. 2A, B), or between those of *HRGP^cre^Gjd2^fl/fl^* mice and the other control genotypes (*Gjd2^+/+^*, *HRGP^cre^Gjd2^+/+^*; see Supplementary Fig. S2).

Two different protocols were used to investigate the light-adapted ERG. In protocol 1,^7^ mice that had been dark-adapted and then tested for scotopic ERG responses were exposed to a steady background adapting light (40 cd/m^2^) inside the ganzfeld. Superimposed upon this background, bright light flashes (0.9 log cd.s/m^2^) were presented at a frequency of 0.75 hertz (Hz) during an 18-minute period of light adaptation. There were no consistent differences in a-wave amplitude between genotypes or time of day (Fig. 2C). In floxed control mice (*Gjd2^fl/fl^*), a circadian rhythm of b-wave amplitude was observed, with higher amplitudes during the subjective day than the subjective night (Fig. 2D), similar to that observed previously for wild type mice.^7^^,^^17^ The mice showed gradual increases in b-wave amplitude as a function of light adaptation time in either the day or the night, but the amplitudes were greater during the daytime (Fig. 2). In contrast, *HRGP^cre^Gjd2^fl/fl^* mice showed greatly reduced day-night difference and little or no light-adaptation time-dependent increase in b-wave amplitudes; the b-wave amplitudes were comparable to those of daytime controls that were fully light adapted (Fig. 2D). Similar observations were made with comparisons to the *Gjd2^+/+^* and *HRGP^cre^Gjd2^+/+^* control mice (see Supplementary Fig. S3A, B). These findings suggest that the *HRGP^cre^Gjd2^fl/fl^* mice were fully light adapted at the beginning of the flash series.

**Figure 3.**
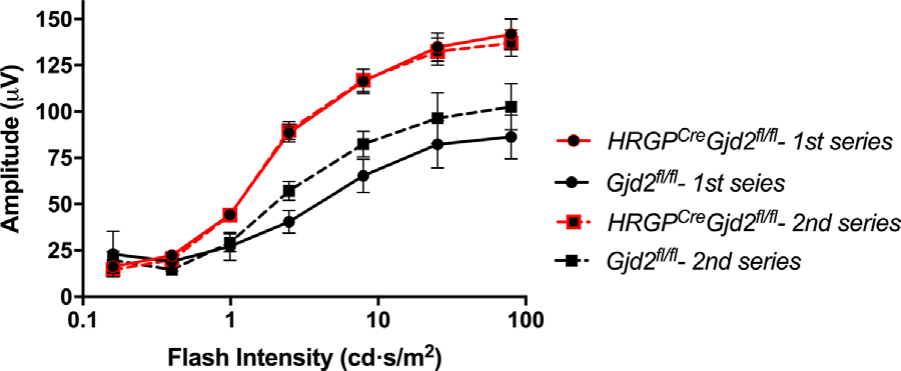
Repeat luminance-response analysis of photopic ERG in *HRGP^cre^Gjd2^fl/fl^* and *Gjd2^fl/fl^* mice. Light-adapted ERG recordings followed protocol 2, and were recorded between ZT 16 and 20. In the control genotypes, the b-wave amplitudes elicited during the second intensity series were consistently higher than those of the first series. In contrast, the first and second intensity series yielded identical b-wave amplitudes in the HRGP*^cre^* Gjd2*^fl/fl^* mice, and these amplitudes were significantly higher than those of the control mice. Significant effects of flash intensity, genotype, and interaction (*P* < 0.01). Sample sizes: *HRGP^cre^Gjd2^fl/fl^* 18 mice; *Gjd2^fl/fl^* 8 mice. Comparisons of recordings from *HRGP^cre^Gjd2^fl/fl^* mice with wild type and *HRGP^cre^Gjd2^+/+^* control mice are shown in Supplementary Figure S3.

In light-adapted ERG protocol 2, dark-adapted mice received a single bright flash (25.3 cd.s/m^2^) in the middle of the subjective night followed immediately by application of the steady background adapting illumination. One minute later, the mice were exposed to a series of flashes of increasing intensity (0.16–79.65 cd.s/m^2^; 25 ms duration; 20 flashes / intensity). One minute after the first intensity series ended, it was repeated, with the mice exposed to the background illumination the entire time. In the control genotypes, the b-wave amplitudes elicited during the second intensity series were always higher than those of the first series, consistent with time-dependent light adaptation (Fig. 3; see Supplementary Fig. S3C, D). In contrast, the first and second intensity series yielded identical b-wave amplitudes in the *HRGP^cre^Gjd2^fl/fl^* mice, and these amplitudes were significantly higher than those of the control mice. These results support the conclusion that the *HRGP^cre^Gjd2^fl/fl^* mice were fully light adapted at the first time-point after the background illumination was applied.

### Retinal Structure in Mice with Cone-Specific Disruption of *Gjd2*

**Figure 4.**
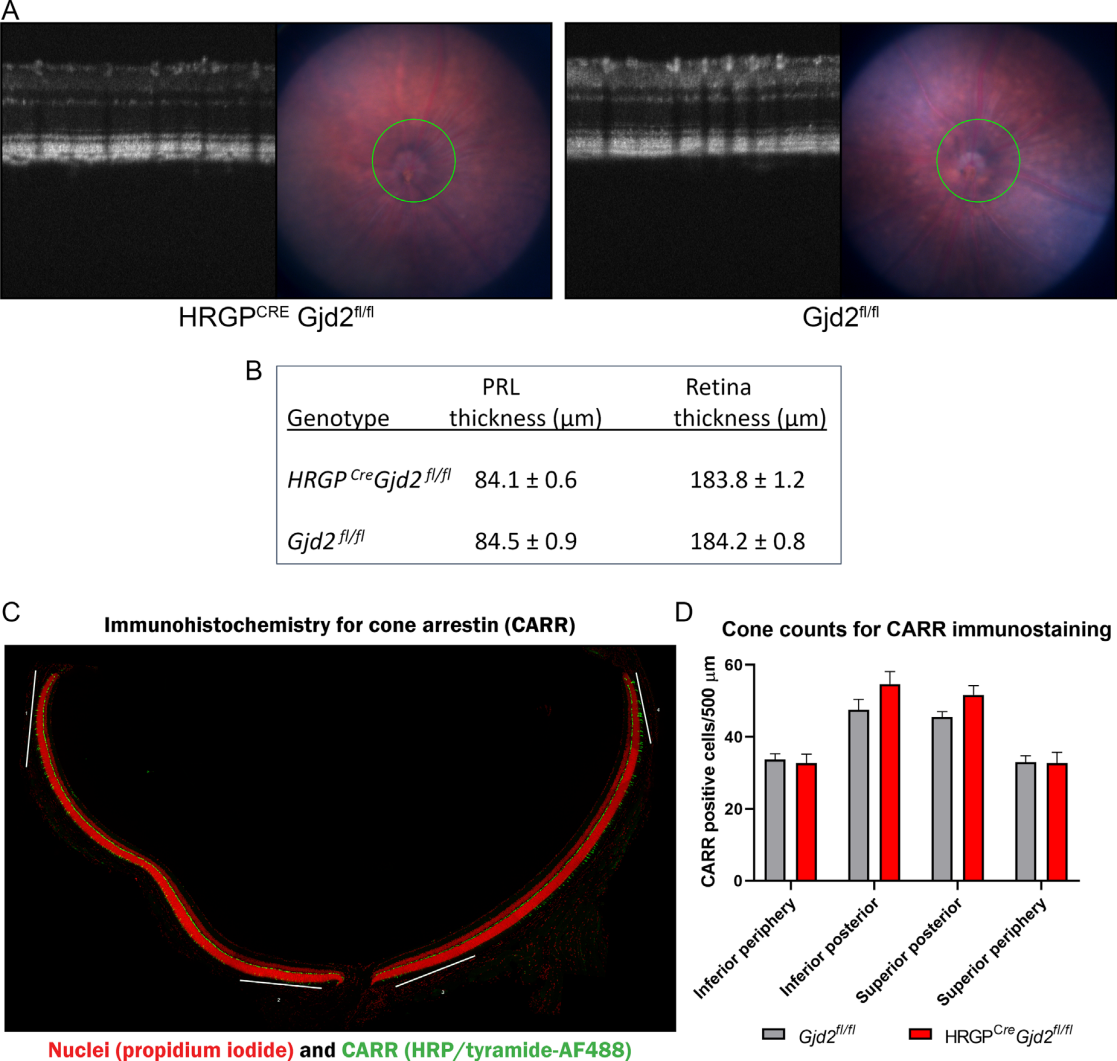
Morphological assessment of retinas of *HRGP^cre^Gjd2^fl/fl^* and *Gjd2^fl/fl^* mice. (**A**) No differences were observed in fundus or SD-OCT images. (**B**) Photoreceptor layer (PRL) thickness and retinal thickness, derived from the SD-OCT images, showed no difference between genotypes; sample sizes: 7 to 9 mice / genotype. (**C**) Representative immunostaining for cone arrestin (CARR; arrestin-4); the white bars approximate the 500 µm areas where CARR positive cells were counted (magnification: 60×). A high power (60×) image of CARR staining is shown in Supplementary Figure S4. (**D**) Quantification of CARR positive cells showed no difference between genotypes in either central or peripheral retina; sample size: 6 retinas from 3 mice of each genotype.

Fundus photographs and SD-OCT were used to obtain initial assessments of retinal structure in the *HRGP^cre^Gjd2^fl/fl^* mice (Fig. 4A). The fundus photographs and OCT images of the *HRGP^cre^Gjd2^fl/fl^* mice and *Gjd2^fl/fl^* controls were not different. Measurements of photoreceptor layer (PRL) thickness and total neural retinal thickness showed no significant differences among genotypes (Fig. 4B). To further explore the possible effect of cone photoreceptor Cx36 removal on cone survival, we counted the number of CARR-4 immunopositive cones in the *HRGP^cre^Gjd2^fl/fl^* mice and *Gjd2^fl/fl^* control mice (Fig. 4C,D; see Supplementary Fig. S4). No genotype differences were observed in either the central or peripheral retina.

## Discussion

When wild type mice are exposed to a steady, rod-saturating background illumination, photopic ERG responses gradually increase as a function of light-adaptation time.^6^^,^^17^ This occurs both day and night, but superimposed on this light-adaptive response is a circadian rhythm of b-wave amplitude, with higher amplitudes during the subjective day than subjective night. This rhythm is reduced in amplitude or eliminated in mice lacking retinal dopamine or clock genes.^5^^–^^7^ The current study investigated the potential role of Cx36 in cone photoreceptors in the light-adaptive response and the circadian rhythm of photopic b-wave amplitude. The results indicate that Cx36 contributes to both the ERG circadian rhythm and to the light-adaptive response to background illumination.

Cx36 in cone photoreceptors contributes to the formation of gap junction between adjacent cones and between rods and cones (reviewed in Bloomfield and Volgyi 2009^18^ and O'Brien and Bloomfield, 2018^12^). Rod-cone coupling via Cx36-containing gap junctions is regulated by light and circadian clocks^19^^,^^20^ by a mechanism that appears to involve dopamine and adenosine coregulation of Cx36 phosphorylation.^21^

Similar to results in wild type mice,^17^ our study showed circadian regulation of photopic b-wave amplitudes of *Gjd2* control mice (*Gjd2^fl/fl^*; *HRGP^Cre^Gjd2^+/+^*; and *Gjd2^+/+^*) with higher b-wave amplitudes during the subjective day than the subjective night. However, the circadian and light-adaptation responses were abnormal in *HRGP^cre^Gjd2^fl/fl^* mice, which have a selective reduction in Cx36 in cone photoreceptors. Using a protocol in which bright light flashes (0.9 log cd.s/m^2^) were superimposed upon the steady background during the subjective day and subjective night, the b-wave amplitudes of *HRGP^cre^Gjd2^fl/fl^* mice were consistently higher than those of controls from the earliest flashes, and there was little day-night difference. These finding suggest that the *HRGP^cre^Gjd2^fl/fl^* mice were fully light adapted as soon as the background illumination was applied or shortly thereafter and that rod-cone or cone-cone gap junctions are required for expression of the circadian rhythm. Alternatively, the higher amplitude responses could have been due to enhanced sensitivity of the cones of *HRGP^cre^Gjd2^fl/fl^* mice. To further explore the possibility that the mice were fully light adapted shortly after application of the background, we used a second protocol in which mice were exposed to a series of light-intensities (0.16–79.65 cd.s/m^2^) beginning 1 minute after application of the background, followed by a second intensity series beginning 1 minute after completion of the first one. In control mice, the second series showed higher b-wave amplitudes than the first series, indicative of time-dependent light adaptation. However, in the *HRGP^cre^Gjd2^fl/fl^* mice, the two intensity-response functions were identical and the amplitudes were dramatically higher than those of controls at all intensities at or above 1 cd.s/m^2^. The half-maximal response of the *HRGP^cre^Gjd2^fl/fl^* mice and controls were similar (approximately 10 cd.s/m^2^), suggestive of no change in sensitivity. These findings suggest that eliminating rod-cone coupling disrupts the ERG circadian rhythm, with b-wave amplitudes in the day and the night locked in the daytime, light-adapted state.

Based on our results with the cone-specific Cx36 disruption, we propose the following working hypothesis for the role of rod-cone gap junctions in regulating the photopic ERG response (Supplementary Fig. S5). Rod-cone gap junctions are open in darkness and in dim light due to high levels of intracellular cyclic AMP and protein kinase A-mediated Cx36 phosphorylation,^22^ allowing rods to signal through cone pathways^23^ (Supplementary Fig. S5A). When the rod saturating background is first applied, the cone currents generated from the flashes are partially shunted into rods, resulting in a relatively small photopic ERG response (Supplementary Fig. S5B). As dopamine release from amacrine cells is stimulated by the background light, dopamine D4 receptor activation on photoreceptors increases, which decreases cyclic AMP levels^24^ and promotes protein dephosphorylation.^25^ The gradual dephosphorylation of Cx36 results in inhibition of the gap junction conductance between cones and rods, electrotonically isolating the cones, increasing their synaptic strength, and, consequently, the amplitude of the photopic b-wave (Supplementary Fig. S5C). This mechanism could account for the light-adaptation time-dependent increase in the photopic ERG response. We further propose that the circadian rhythm in b-wave amplitude is mediated, at least in part, by circadian expression of the dopamine D4 receptor.^26^^,^^27^ The absence of Cx36 in the cones of *HRGP^cre^Gjd2^fl/fl^* mice mimics the effects of dopamine-mediated rod-cone gap junction closure, resulting in the stronger ERG response as soon as the background light is applied and the cones are stimulated with flashes, because the cone currents are not shunted into rods (Supplementary Fig. S5D).

Thus, our data suggest that rod-cone gap junctions are not only important for rod signaling through cone pathways, but that their regulation also contributes to network adaptation in the cone pathway under photopic conditions and to the circadian regulation of photopic ERG responses.

## Supplementary Material

Supplement 1

Supplement 2

Supplement 3

Supplement 4

Supplement 5
