# Supplementary material for: Effects of Cone Connexin-36 Disruption on Light Adaptation and Circadian Regulation of the Photopic ERG
Source: Invest Ophthalmol Vis Sci. 2020 Jun 12;61(6):24. doi: 10.1167/iovs.61.6.24 (PMC7415284; doi:10.1167/iovs.61.6.24)
Supplement: Supplement 1 [file iovs-61-6-24_s001.pdf]

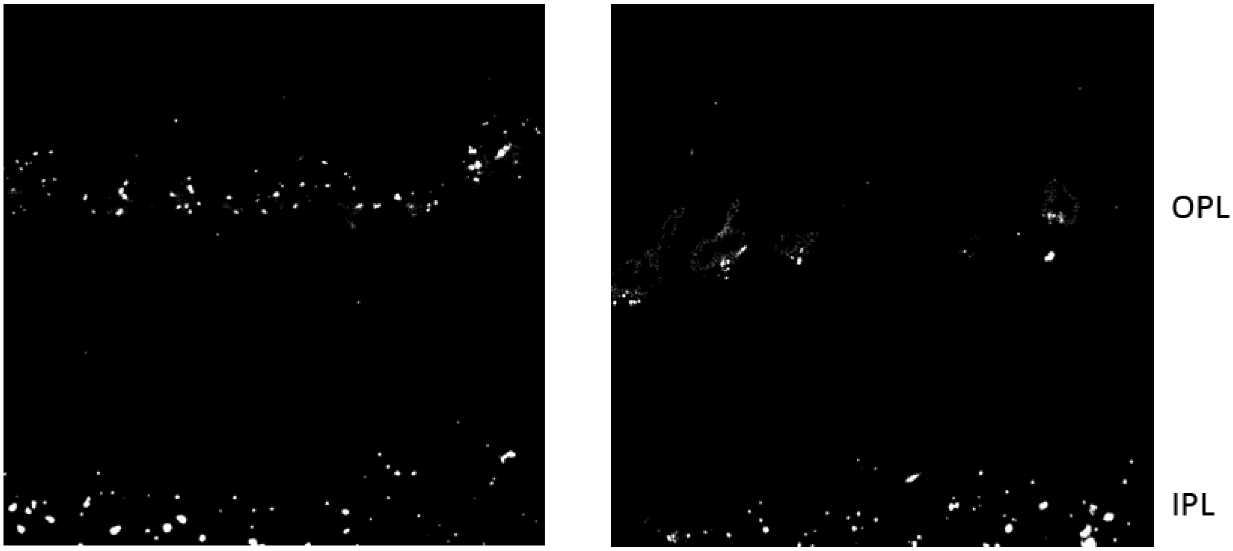

Gjd2$^{fl/fl}$ *HRGP-Cre Gjd2*$^{fl/fl}$

Supplemental Figure S1. Black and white images of Cx36 immunostaining in *Gjd2*$^{fl/fl}$ and *HRGP*^{cre}*Gjd2*$^{fl/fl}$ mouse retinas. The images (240X) from Figure 1 were converted to black and white. Note the lower level of staining in the outer plexiform layer (OPL) but not the inner plexiform layer (IPL) of *HRGP*^{cre}*Gjd2*$^{fl/fl}$ retina compared to the *Gjd2*$^{fl/fl}$ control sample.
