# Supplementary material for: Effects of Cone Connexin-36 Disruption on Light Adaptation and Circadian Regulation of the Photopic ERG
Source: Invest Ophthalmol Vis Sci. 2020 Jun 12;61(6):24. doi: 10.1167/iovs.61.6.24 (PMC7415284; doi:10.1167/iovs.61.6.24)
Supplement: Supplement 2 [file iovs-61-6-24_s002.pdf]

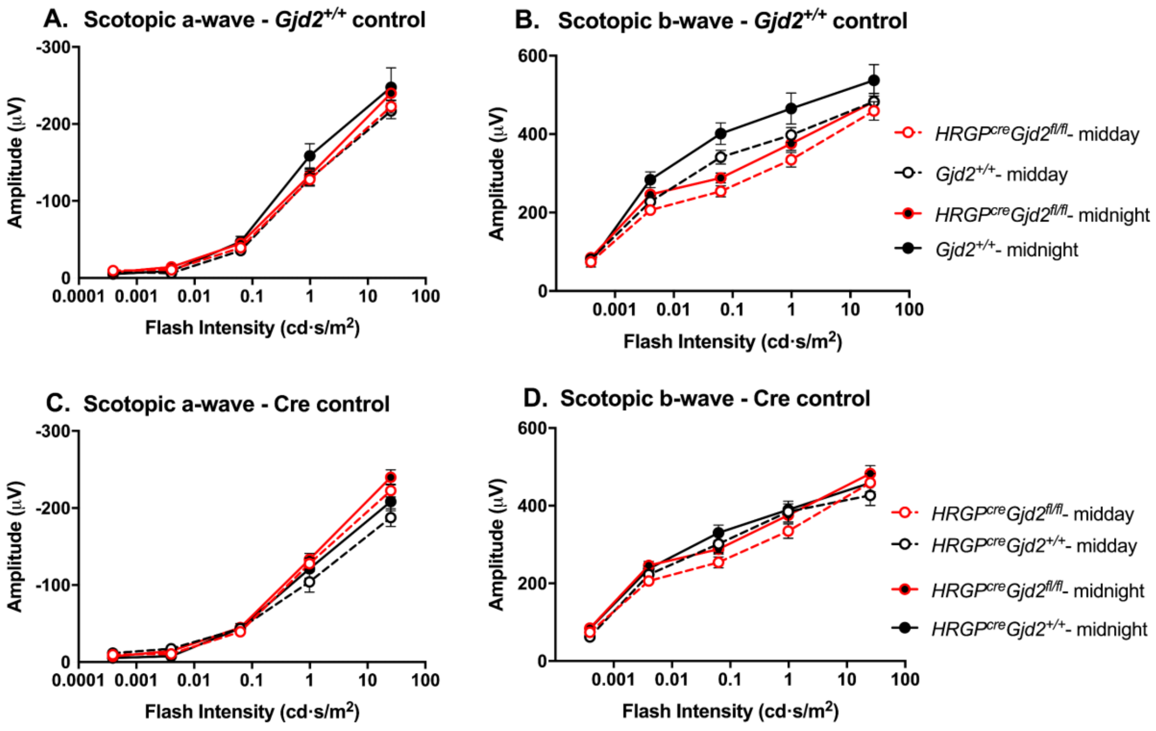

Supplemental Figure S2. Scotopic ERG recordings of $HRGP^{Cre}$ $Gjd2^{fl/fl}$ and controls. (A, B) No differences with dark-adapted ERG a- and b-wave amplitudes between $HRGP^{Cre}$ $Gjd2^{fl/fl}$ mice and $Gjd2^{+/+}$ control mice. (C, D) No differences with dark-adapted ERG a- and b-wave between $HRGP^{cre}$ $Gjd2^{fl/fl}$ mice and $HRGP^{Cre}Gjd2^{fl/fl}$ control mice. Sample sizes: $HRGP^{cre}Gjd2^{fl/fl}$ 18 mice; $Gjd2^{fl/fl}$ 8 mice; $Gjd2^{+/+}$ 7 mice; $HRGP^{cre}$ $Gjd2^{+/+}$ 11 mice. The data for $HRGP^{cre}Gjd2^{fl/fl}$ and $Gjd2^{fl/fl}$ mice were replotted from Figure 2.
