# Supplementary material for: Effects of Cone Connexin-36 Disruption on Light Adaptation and Circadian Regulation of the Photopic ERG
Source: Invest Ophthalmol Vis Sci. 2020 Jun 12;61(6):24. doi: 10.1167/iovs.61.6.24 (PMC7415284; doi:10.1167/iovs.61.6.24)
Supplement: Supplement 3 [file iovs-61-6-24_s003.pdf]

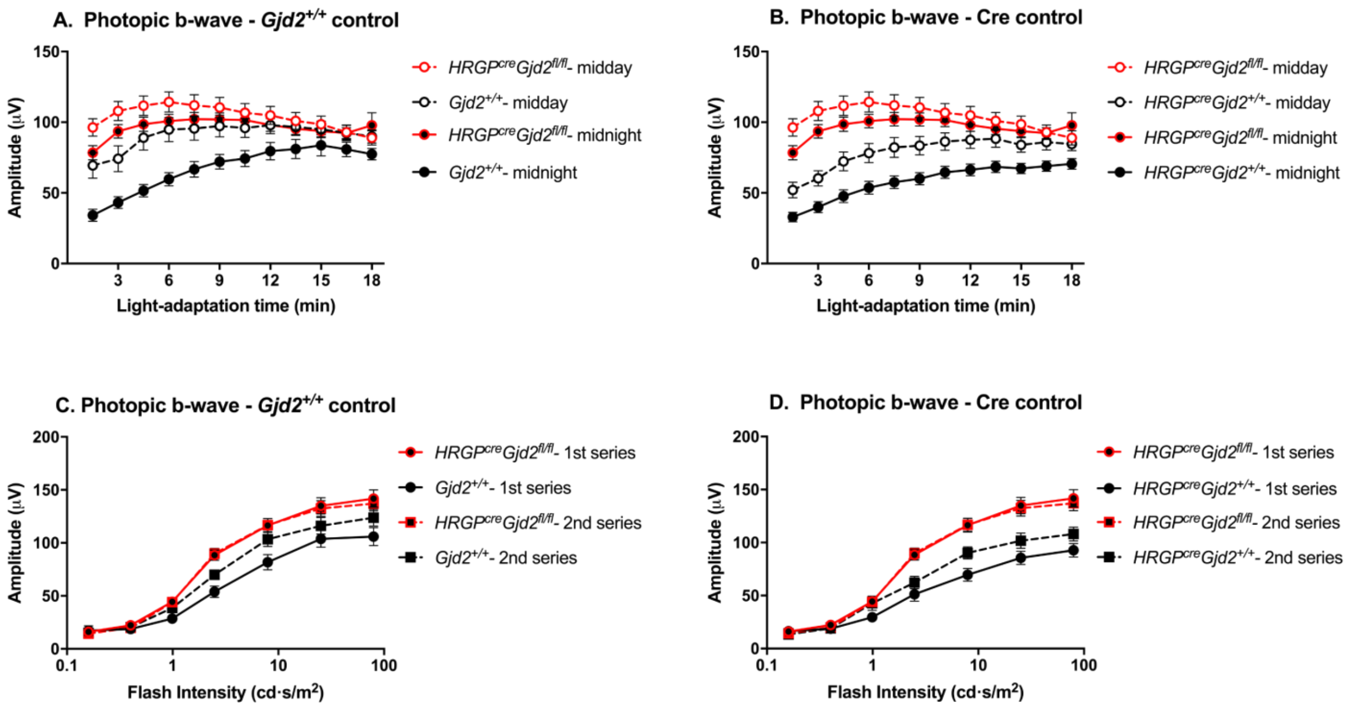

Supplemental Figure S3. Photopic ERG recordings of *HRGP^{cre}Gjd2$^{fl/fl}$* and controls. (A, B) *HRGP^{cre}Gjd2$^{fl/fl}$* mice had constitutively higher photopic b-wave amplitudes compared to controls at all light-adaptation times up to 12.5 min, and had reduced midday-midnight differences compared to controls. (C, D) In the controls, the b-wave amplitudes elicited during the second intensity series were consistently higher than those of the first series; the first and second intensity series yielded identical b-wave amplitudes in the *HRGP^{cre}* Gjd2$^{fl/fl}$ mice, and these amplitudes were significantly higher than those of the control mice ($p<0.05$). Sample sizes: *HRGP^{cre} Gjd2$^{fl/fl}$* 18 mice; *HRGP^{cre} Gjd2$^{+/+}$* 11 mice; Gjd2$^{+/+}$ 7 mice. The data for *HRGP^{Cre}Gjd2$^{fl/fl}$* mice were replotted from Figures 2 and 3.
