# Supplementary material for: Effects of Cone Connexin-36 Disruption on Light Adaptation and Circadian Regulation of the Photopic ERG
Source: Invest Ophthalmol Vis Sci. 2020 Jun 12;61(6):24. doi: 10.1167/iovs.61.6.24 (PMC7415284; doi:10.1167/iovs.61.6.24)
Supplement: Supplement 4 [file iovs-61-6-24_s004.pdf]

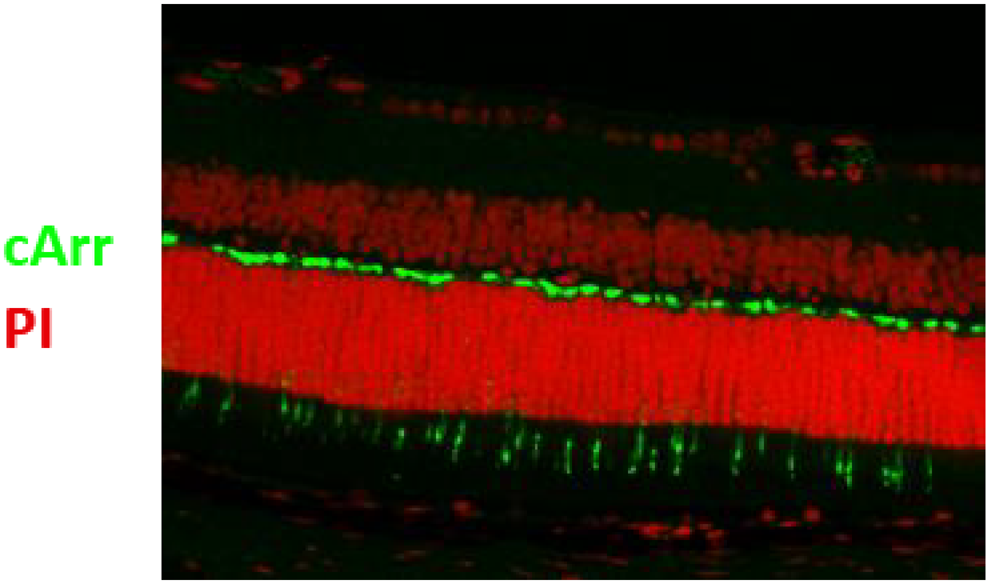


**Supplemental Figure S4. Representative cone arrestin (CARR; green) staining of *HRGP*$^{cre}$*Gjd2*$^{fl/fl}$ mouse retina.** Counter stained with propidium iodide (PI; red). Photographed at 60X.
