# Supplementary material for: Effects of Cone Connexin-36 Disruption on Light Adaptation and Circadian Regulation of the Photopic ERG
Source: Invest Ophthalmol Vis Sci. 2020 Jun 12;61(6):24. doi: 10.1167/iovs.61.6.24 (PMC7415284; doi:10.1167/iovs.61.6.24)
Supplement: Supplement 5 [file iovs-61-6-24_s005.pdf]

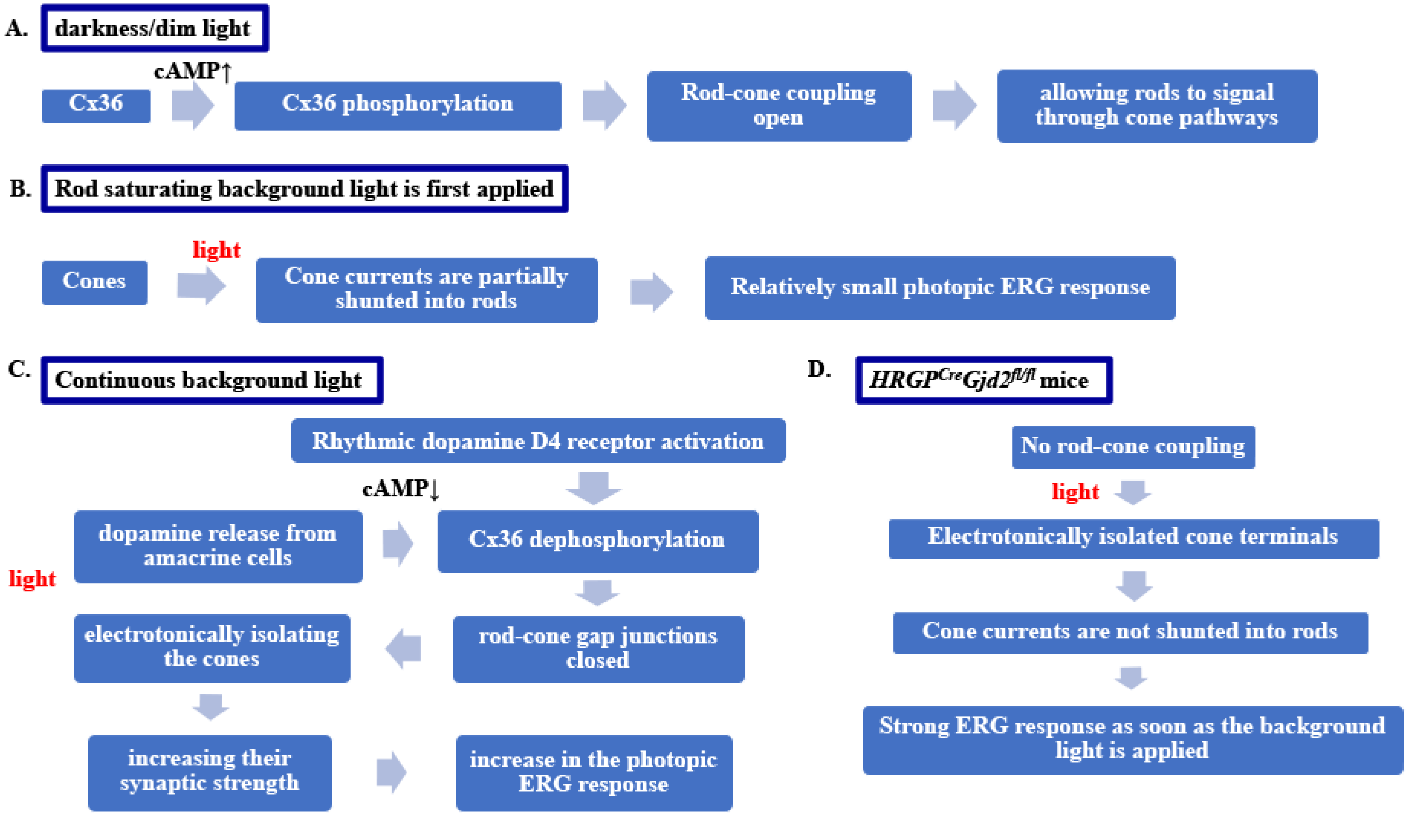

Supplemental Figure S5. Working hypothesis for the role of Cx36 in the regulation of cone responses. See the Discussion for details.
